# Foreign

**Published:** 1842-02-05

**Authors:** 


### FOREIGN.

*Clinical Remarks by* Dr. Marshall Hall, *on the Use of Setons.*

There is no doubt that the seton is a most valuable remedy; at the same time, it is a most disagreeable one, and few patients submit to it willingly. The circumstances which render it advisable are, we believe, a chronic disease in an organ more or less inflammatory, and generally attended with a purulent or other discharge. In such cases, the seton is one of the most powerful and, of course, permanent revulsives we possess. But there is one condition which is almost essential for its favourable action, that is, that the general health and state of the individual should be maintained; hence we rarely prescribe setons, except in conjunction with tonics, and a more or less nourishing diet. The alterative tonics are the best, such as the preparations of iodine, sarsaparilla, &c.

The careless use of setons is, however, productive of so much mischief, that we are not nearly so much accustomed to use them as many of our brother practitioners. When the patient is losing his strength and flesh; when he is of a nervous, excitable temperament, setons are almost always mischievous; and even if the conditions of the patient be not, in all respects, such as to forbid the use of the seton, still, they are sometimes used, absurdly enough, for mere nervous functional disturbances, often depending on disorders of a neuralgic nature, which require a treatment totally different from that of setons. In tuberculous diseases setons are evidently injurious, if the general health of the patient fail; and in the mere lesions, such as cicatrices of the brain, which remain after apoplexy, they are absurd, unless a positive action continues in the cerebral substance: if such should be the case, setons are often of service.—[*Eds. Ex.*]

Many years ago, I was consulted by Mr. Doubleday, of Blackfriars Road, in the case of a young married lady who had suffered from peritonitis after her first accouchment.

This peritonitis appeared to be confined to the pelvic region. Its acute character had been subdued, but tenderness, with tumidity, and difficulty in voiding the bladder and rectum remained. I made a careful examination. A distinct hardness was felt under the pubes, extending to one side, I think the left. On examination per vaginam and per rectum, a similar hardness was found occupying the lower part of the pelvis. I imagined this hardness to consist in coagulable lymph, effused from the inflamed peritoneal surfaces of the pelvis, producing the symptoms by its pressure on the neck of the bladder and on the rectum.

We strictly regulated the diet and the intestines, and inserted an ample seton over the induration. Slowly and gradually that induration, with its attendant symptoms, became diminished, and eventually diappeared.

Several years after this, I was consulted in the case of the sister-in-law of this patient, under very nearly similar circumstances. The same remedy was followed by the same happy result.

A year ago I was consulted by Mr. Burford, in the case of a gentleman of sixty, who had become affected with pain, tenderness, and tumidity of the abdomen. On a careful examination, a distinct hardness was felt in the midst of the general tumidity, occupying the region of the caput cœcum coli. We regulated the diet and the bowels, administered mercury, and inserted an ample seton. The mouth became affected, and the seton discharged copiously: the hardness and the other symptoms gradually, but at length entirely disappeared.

A similar case occurred a year ago, in the person of a gentleman of forty, a patient of Mr. Squibb, in Orchard street. A strict regimen was enjoined, the bowels regulated, and an ample seton was inserted. The induration, which in this case occupied the space between the false ribs and the ilium, on the left side, gradually disappeared.

Two years ago I was consulted by Mr. P———, a barrister, affected with pneumonia of the middle and upper lobes of the right lung. A seton was inserted, and Mr. P——— went to Maderia. On his return, the physical signs and the symptoms of the pneumonia had disappeared.

I have still more recently treated a case of pneumonia of the upper portion of the right lung, in consultation with Mr. Beane of Peckham. A seton was inserted, and in six weeks a most decided amendment in the physical signs, the symptoms, and the general health occurred. Since that period the patient has continued to improve, and now no dulness on percussion, or other signs of disease, is perceptible.

In a variety of cases of acute or chronic, local or limited internal inflammation, I have had recourse to the seton, and uniformly with the most marked success; so that, I think, we may look upon the remedy as almost specific in such cases. It is unnecessary to enumerate them. But hepatitis and nephritis belong to them in an especial manner, and I would suggest this remedy as likely to be of service (if any remedy can) in the case of albuminous urine. In one such case the urine was more albuminous after cupping. I imagined [the] effect arose from the mechanical violence inflicted, and recommended the cupping to be performed above and below the precise region of the kidneys. Under the use of this remedy the albumen diminished, and even ceased for a time.

These and other cases, then, induce me to think that there can be little doubt of the real efficacy of the seton in chronic inflammation. The object is to demonstrate this in some measure, and then to notice briefly farther applications of the remedy. I do not pretend to suggest any thing new, but rather to enforce what is old. The efficacy of setons, when appropriately applied in the nucha, (for they are frequently employed very uselessly,) is well known. The proper cases are inflammation and congestion. But the case to which I would particularly draw attention is that of disease of the spinal marrow, with paraplegia, or paraplegic spasm.

In this case issues are generally inserted. They appear to me far more painful, far less manageable, and far less efficacious than ample setons. They have also, I am persuaded, been generally applied below the real seat of the disease. I was consulted a few weeks ago by a gentleman from Manchester. With partial loss of power, he had loss of sensation in the lower extremities; the numbness extended to a line just above the sacrum. Issues had been applied on each side of this line. They might, with equal efficacy, have been applied to the foot! I need not say that the spinal nerves procceed, for some distance. from above directly, rather than obliquely, downwards, and that the seat of the disease is *at* or ABOVE their junction (insertion or origin) with the spinal marrow.

Bearing these two principles in mind, then, viz. that ample setons afford a more efficacious counter irritation than issues, and that they ought to be applied higher along the spinal column than has been usual, I think we have a new mode of treatment for this formidable class of diseases.

These setons should, besides, be larger than usual. They should be three-fourths of an inch in breadth, and extend through two inches in length, be inserted on the level with and above the supposed seat of the disease, (the anatomy being consulted) and be four or six in number, two or three being instituted on each side of the spinal column. Acting on this principle, I had, five days ago, the pleasure to receive the most satisfactory account of a patient affected with paraplegia, whom I had seen at Lohan, in Essex, in consultation with Mr. Gross.

I repeat, and beg to conclude by repeating, that I believe counter-irritation applied along the spine has failed, because it has been applied below the seat of the disease; and that, to be efficacious, it must be both more efficient in itself, and applied with greater regard to the anatomy of the spinal marrow and nerves. The precise spot for their application must be left to the well-informed practitioner. I need scarcely remind my reader, that the persistence or cessation of all reflex actions, will determine whether our remedies should be applied above or below the origin of the cauda equina, above or below the last dorsal vertebra.

We are great advocates for setons too. We confess we have not used them so broad nor so many at a time as our friend Dr. Hall. But perhaps he is right. The difficulty would be, in some cases, to induce patients to submit to their introduction.

We have tried setons in three or four cases of albuminous urine. They were bad cases certainly, and the effect was not encouraging.—*London Medico-Chirurgical Review, from London and Edinburgh Journal of Med. Science, No.* 11.

---

### *Observations on the Nature and Treatment of Various Diseases.* By ROBERT J. GRAVES, M. D.

### REMARKABLE AND UNEXPECTED RECOVERY FROM LARGE ABSCESSES OF THE LUNGS.

Though the introduction of the stethoscope has been of the greatest utility in the investigation of pulmonary complaints, both as regards their prognosis and their treatment, it must be confessed that, in many instances, practitioners have been induced unduly to rely upon the indications of disease which this instrument affords, and consequently have seen their prognosis fail. The following remarkable cases afford abundant proof, that patients may recover, contrary to the usual interpretation of the most significant and decisive stethoscopic symptoms, and therefore seem to merit publication, in order to warn practitioners from relying too exclusively upon physical phenomena, and too hastily concluding that pulmonary lesions, however extensive, thus indicated, must necessarily prove fatal. These cases, too, show that vast abscesses may be formed in the lungs, and yet the patient recover; and likewise, that real circumscribed abscess occurs more frequently in the pulmonary tissue than Laennec allowed, or his followers seem to believe. It is true, indeed, that where suppuration takes place in the lung, nature effects it in a manner either calculated to afford the readiest exit for the matter so formed, or best suited to promote its absorption.

This object, from the extent of the parenchymatous structure of these organs, and its relation to the air cells and minute bronchial tubes, is most easily effected, by so disposing of the purulent fluid, resulting from inflammation, that it can, on the one hand, be with facility eliminated through the bronchial tubes, or on the other absorbed in the texture of the lung itself. In other organs and other parts, a similar facility for mechanical elimination does not exist, and consequently the easiest step which nature can take is, to collect the puriform fluid, within the parietes of a circumscribed abscess, which may work its way outwards for the purposes of discharge. From this view it appears, that in other parts, circumscribed abscess is the ordinary means of evacuation provided by nature, and diffuse suppuration the exception; while in the lungs the reverse obtains, diffuse suppuration being the ordinary rule, and circumscribed abscess the exception. The rationale here exposed has been well explained by Dr. Stokes, in his admirable treatise on diseases of the lungs, but at the time he wrote, neither he nor I were aware that large abscesses occur so frequently in the lungs, or are so often recovered from, as subsequent observation has shown to occur.

Case I.—In the year 1837, I was called to visit a boy at Rathmines, who presented the following symptoms: he had for many weeks been affected with cough, dyspnœa, and bloody expectoration, attended by fever, emaciation, and colliquative sweats; and when I saw him his pulse was extremely quick, his respiration hurried and difficult, while his whole appearance expressed danger of almost immediate dissolution.

The right side of his chest, but more particularly the superior part below the clavicle, was dull on percussion, and every time he coughed, matter could be heard gurgling in a vast cavity in the upper part of the lung; the gargouillement was so plain as not to require the application of the stethoscope, and indeed it was almost impossible for even the most zealous cultivator of science to examine the physical phenomena very closely, for every time he coughed he threw up large quantities of purulent matter, mixed with blood of a stench so insufferable that my stomach was nauseated, and I could not remain more than a few minutes in his room, even the most distant parts of which were pervaded by this abominable fœtor. I at once pronounced the case hopeless, and advised merely palliative treatment. In a few months afterwards, I was surprised to see the same boy apparently recovered, assisting in carrying on his father's business, that of a tavern-keeper. He has since grown up and become a tolerably strong young man, healthy in every respect, except a certain degree of shortness of breath, which he feels when forced to make any considerable exertion. A manifest flattening is still evident beneath the right clavicle.

Case II.—In the summer of 1839, Sir Philip Crampton brought me to the Shelbourne Hotel, to see a boy about twelve years of age, who had been at school in France, and had caught a cold in the preceding spring, under the effects of which he had ever since laboured. The disease had been but little attended to, and no appropriate treatment employed until emaciation had considerably advanced, and his constitution was evidently sinking under the inroad of the malady. His father was then written to, and he proceeded in haste to the school, where he found that an eminent physician had pronounced the boy's case hopeless, and had declared that he was in the last stage of phthisis. He was brought to Ireland by short stages, and though his removal was accomplished with all due care and circumspection, yet his parent was more than once in a state of well-founded apprehension that he would expire on the road. The disease in this case had been *so long in forming*, *had advanced so steadily*, and had attained to such a degree of intensity, that little or no hope remained of his recovery. The physical phenomena and the constitutional affection were much the same as those detailed in the preceding case, with the exception that the expectorated pus was neither so abundant nor so fœtid. In both this case and the preceding, it is to be remarked, that only one lung was affected. His parents were anxious to remove him to the country, and Sir Philip Crampton and I felt much hesitation in sanctioning this step, as the danger of his immediate dissolution was so imminent His friends, aware of this danger, nevertheless executed their intention; and about five months afterwards I was astonished to learn that the boy had perfectly recovered, and was then engaged in frequently enjoying the diversion of hunting in the County Tipperary.

In both these young persons, the history of the disease, and its unexpected termination, prove that they were affected with chronic pneumonia, ending in the formation of vast abscesses in the upper portion of the lung, which brought both patients into a state of the greatest jeopardy, but finally yielded to the curative powers of nature.

I do not see how, in either, a physician was to distinguish them from tubercular abscess. Had the disease in either been more acute the diagnosis might have been possible; but, in both, its progress was at first insidious, occupying many months previous to the formation of the cavities, and accompanied by gradually increasing constitutional symptoms and hectic fever. The mere freedom of one lung from disease does not constitute a certain means of diagnosis, for the same not unfrequently obtains in true tubercular phthisis. In such cases, it is probable, that the microscopical examination of the expectorated fluid would have thrown important light on the subject, and have revealed the true nature of the disease, but it is only lately that investigation has been directed to this promising field of inquiry, on which Dr. Watts has in this Journal made several excellent remarks.

Case III.—Early in the spring of 1841, Dr. Brereton brought me to see, at Sandford, a young boy about fourteen or fifteen years of age, who a fortnight before had been attacked with symptoms of pleuro-pneumonia, intense pain in the side, and cough of a very harassing character; he had also expectorated considerable quantities of the characteristic sputa, tinged of a prune juice colour. The constitutional symptoms had all along been very severe, and together with the local inflammation, had not yielded to a very active and judicious treatment. For about ten days after my first visit, matters went on from bad to worse, and at the end of that time, his pulse was about 140; dyspnœa excessive; uneasiness, jactitation, and restlessness; constantly urgent cough, both night and day, so that his case appeared utterly hopeless, and his death was momentarily expected. The pneumonia occupied nearly the whole of the right lung, and rendered that side almost every where dull; and during the first periods of the disease, crepitus had been extensively present. While matters thus threatened a speedy and unfavourable termination, he was seized at night with intense difficulty of breathing, anxiety and pain in his side, and seemed to be moribund. With a sudden effort, however, he succeeded in expectorating a very large quantity of purulent matter, and immediately obtained comparative relief. A similar struggle took place on the following night, and with a similar result, and when I saw him the next morning, I found him in some respects manifestly relieved, but still labouring under great debility, considerable difficulty of breathing, and fever. On examining the right side of chest, the whole anterior portion, from immediately below the clavicle downwards, as far as the bottom of the lung, was found to be morbidly resonant on percussion; a change of a most striking nature, for these parts had been before quite dull. This side of the lung was now evidently dilated, and the stethoscope detected a loud and well-marked metallic tinkling, whenever he coughed or spoke. The detection of this phenomenon rendered it certain that a vast abscess existed in the lung, communicating certainly on the one hand with the bronchial tubes, and not improbably on the other with the pleural cavity; a view of the subject which, in my mind, rendered the case hopeless, and I pronounced it to be so. For a fortnight or longer, he had occasional returns of sudden purulent expectoration, each time, however, less in quantity, and followed by more marked relief of the constitutional symptoms, and about six weeks from the occurrence of the first expectoration of matter, his convalescence had far advanced, and he is now strong and healthy.—*Dub. Journ. Med. Science, Jan.* 1842.

(*To be continued.*)

*Connection between abundance of food and mortality.* By M. Melier.—In this memoir, which was read at the Academy of Medicine of Paris on the 7th of September, the author established, by numerous documents drawn from the histories of various countries, that the number of deaths always correspond to the price of food. "Wherever there's a loaf added, there's a man born," said an economist: and nothing is more true than this metaphorical expression. If we represent the variations of the general mortality and those of the price of bread at different times, by two curved lines which rise and fall with all the fluctuations of these particulars, we shall find all their curvatures exactly, and with the most perfect regularity, corresponding. The constant increase of the population of France for a certain number of years, is easily explained by the progress of agriculture, the modifications which the laws relating to corn have undergone, and especially by the introduction of potatoes. The influence of the dearness of food, however, is observed more distinctly in the year next following than in any in which it has occurred.—*London Med. Gaz., from Gaz. Med.*, September 10, 1841.

---

*Compound dislocation of the thumb—reduction—cure.*—John Williams, aged 18, was admitted an out-patient of the University College Hospital, under Mr. Morton, on the 17th of September, 1841, on account of a compound dislocation of the first phalanx of the thumb. The articular extremity of the first phalanx projected completely through the wound, which was situated on the ulnar side of the joint; the end of the bone was very easily reduced. The accident was caused by accidently thrusting his hand with great violence against a wall. The bone being replaced, the end of the bone was retained by several slips of plaster neatly applied, and a small splint of pasteboard was placed underneath the whole length of the finger, so as to support the bones composing it; a bandage was then applied over the whole. To have five grains of calomel and fifteen of jalap directly: to be placed on low diet, and cold lotion to be applied to the part.

This man presented himself at the visit of to-day; but as the parts looked well, the plasters and splints were allowed to remain, while the bandage was removed.

30. Doing very well; wound is almost healed; bandage removed. An abscess having formed upon the under surface of the ball of the thumb, it was opened, and its contents discharged.

Oct. 4. Wound perfectly healed. To use passive motion of the joint.

20. The motions of the joint are preserved. Discharged, not quite cured.—*London Lancet, January* 1, 1842.

---

*The Medical Examiner* is published every Saturday by *J. C. Turnpenny*, N. E. corner of Spruce and Tenth streets. Terms—*three dollars* a year for one copy, or *five dollars* for two copies sent to the same post office, in all cases in advance. Postage the same as on newspapers. Letters to be addressed,—post-paid,—to the Editors of the Medical Examiner, Philadelphia.
*Reynell Coates, M. D.*, Acting Editor.
*J. B. Biddle, M. D.*, and *W. W. Gerhard, M. D.*, Co-editors.
*J. B. Biddle, M. D.*, Proprietor.

